# Supplementary material for: Origin of wiring specificity in an olfactory map revealed by neuron type–specific, time-lapse imaging of dendrite targeting
Source: eLife. 2023 Mar 28;12:e85521. doi: 10.7554/eLife.85521 (PMC10195080; doi:10.7554/eLife.85521)
Supplement: Supplementary file 1. — A supplemental table describing the biological and technical variations we observed among individual brain samples, and measures we took to minimize them, if possible. [file elife-85521-supp1.docx]

**Supplementary file 1:**

**Table 1. Sample variability among individual brains**

| **Variability type and source** | **Measures to minimize, if possible** |
| --- | --- |
| **Biological Variations** | |
| Developmental rate: The rate might differ among individual pupae. | To minimize the variation in developmental rate, we followed an established protocol from our early studies (Jefferis et al., 2001), collected 0–3h APF white pupae, and raised them until indicated developmental stages.  We utilized two independent binary systems to co-label different PN types in the same brain. This approach minimizes technical and biological variations, allowing us to draw direct comparisons without the need of a ‘standard’ brain. |
| Cell number of each PN type: This can vary even in the left and right antennal lobes of the same brain (Bates et al., 2020). | The initial dendrite targeting position appears to be independent of the cell number (compare dendrite targeting of multiple DL1 PNs in **Figure 3A_3_** with that of single DL1 PN in **Figure 3D_3_**). |
| Cell body positions of the same PN type: For example, DL1 cell bodies can be found on either the anterior (**Figure 3D_2_**) or the dorsal (**Figure 3D_3_**) side of the antennal lobe. | The initial dendrite targeting position appears to be independent of the cell body position (compare dendrite targeting in **Figure 3D_2–3_**). |
| **Technical Variations** | |
| Mounting angles | We excluded mounted brains that were twisted, stretched, damaged, or improperly orientated. |
| Staining efficiency | We excluded brains that were stained unevenly (as revealed by the Ncad neuropil staining). |
| Genetic design: Given the random nature of FLP/*FRT* recombination, we speculate that we might only label a subset of neurons of a given PN type, and thus the number of PNs being labeled should vary among individual samples. | As mentioned above, variation in cell number should have no effects on dendrite targeting. |
